# Characterization of the Eukaryotic Virome of Mice from Different Sources

**DOI:** 10.3390/microorganisms9102064

**Published:** 2021-09-30

**Authors:** Chunye Zhang, Matt Burch, Kristine Wylie, Brandi Herter, Craig L. Franklin, Aaron C. Ericsson

**Affiliations:** 1 Department of Veterinary Pathobiology, University of Missouri, Columbia, MO 65211, USA; czvw9@mail.missouri.edu (C.Z.); mmbprd@mail.missouri.edu (M.B.); 2 Department of Pediatrics, Washington University, St. Louis, MO 63110, USA; kwylie@wustl.edu (K.W.); brandiherter@gmail.com (B.H.); 3 McDonnell Genome Institute, Washington University, St. Louis, MO 63110, USA; 4 Metagenomics Center, University of Missouri, Columbia, MO 65201, USA; 5 Mutant Mouse Resource and Research Center, University of Missouri, Columbia, MO 65201, USA

**Keywords:** gut microbiome, virome, laboratory mice, pet store mice, translatability, reproducibility, novel virus

## Abstract

Accumulating studies show that the host microbiome influences the development or progression of many diseases. The eukaryotic virome, as a key component of the microbiome, plays an important role in host health and disease in humans and animals, including research animals designed to model human disease. To date, the majority of research on the microbiome has focused on bacterial populations, while less attention has been paid to the viral component. Members of the eukaryotic virome interact with the commensal bacterial microbiome through trans-kingdom interactions, and influence host immunity and disease phenotypes as a collective microbial ecosystem. As such, differences in the virome may affect the reproducibility of animal models, and supplementation of the virome may enhance the translatability of animal models of human disease. However, there are minimal empirical data regarding differences in the virome of mice from different commercial sources. Our hypotheses were that the mice obtained from pet store sources and lab mice differ in their eukaryotic virome, and that lab mice from different sources would also have different viromes. To test this hypothesis, the ViroCap platform was used to characterize the eukaryotic virome in multiple tissues of mice from different sources including three sources of laboratory mice and two pet stores. As expected, pet store mice harbored a much greater diversity within the virome compared to lab mice. This included an ostensibly novel norovirus strain identified in one source of these mice. Viruses found in both laboratory and pet store populations included four strains of endogenous retroviruses and murine astrovirus with the latter being restricted to one source of lab mice. Considering the relatively high richness virome within different samples from healthy humans, these data suggest that mouse models from alternative sources may be more translational to the human condition. Moreover, these data demonstrate that, by characterizing the eukaryotic murine virome from different sources, novel viruses may be identified for use as field strains in biomedical research.

## 1. Introduction

Animal models, especially mouse models, are used in biomedical research to investigate conditions (including chronic diseases such as autoimmune diseases, cancer, human immunodeficiency virus infection) and acute conditions (such as many infectious diseases). While the advantages of using mouse models are appreciated, there are limitations to be considered in terms of their reproducibility and accurate recapitulation of the human conditions they are used to study [1,2,3,4]. For example, one clinical study using the same drugs that worked very well in experimental treatment in an established mouse model of human amyotrophic lateral sclerosis (ALS) disease [5] was unable to reproduce these preclinical results when applied to a human population [3]. This example of therapeutic failure of a promising drug during clinical trials, combined with other study results [6,7,8] related to the reproducibility or translatability of rodent studies, suggests a need for a more thorough characterization and consideration of the mouse models used in biomedical research.

The bacterial microbiome has gained extensive attention among the biomedical research community because of its influence on many physiological parameters, and association with many adverse health outcomes. For instance, the bacterial gut microbiome is important for metabolism [9], mucosal barrier function [10], defense against certain pathogens [11], and regulation of the immune system [12,13]. Certain compositional features of the microbiome have also been associated with specific diseases such as inflammatory bowel disease [14], colorectal cancer [15] and obesity [16], among others [17]. In contrast to research on the bacterial microbiome, there are relatively few studies focused on the viral portion of the microbiome, i.e., the virome (including prokaryotic and eukaryotic viruses), a fundamental component of the host-associated microbiome [18,19]. Investigations of the eukaryotic virome are hindered by the lack of an efficient technique for untargeted detection of all viral nucleic acid present within a sample, with high sensitivity and specificity.

Previous studies comparing the bacterial gut microbiota of mice from different commercial sources showed significant differences in diversity and composition between the suppliers, and dramatically increased viral pathogen loads in mice obtained from non-traditional sources of mice such as pet stores [20,21,22]. Therefore, in the present study, we hypothesized that pet store mice would harbor a more complex eukaryotic virome than mice from traditional sources of research mice, and that lab mice from different sources would also differ in their virome. We also hypothesized that both sources (pet store and lab) of mice may contain novel viruses.

To characterize the eukaryotic virome (including both DNA and RNA viruses) present in multiple tissues of mice from different sources, weaning age mice were purchased from three commercial suppliers of laboratory mice (Jackson, Taconic, and Envigo) and two local pet stores, and multiple tissues (ileum, perianal skin, and lung) were collected and analyzed with a robust, virus isolation-independent, probe-based targeted nucleic acid enrichment approach, ViroCap [23]. ViroCap is a targeted sequence capture panel containing specific probes that target the complete genomes of 337 viral species and enables the detection of known viruses, as well as novel viruses based on sequence similarity to viral probe sequences. Using this approach, we provided a comprehensive assessment of the virome in these tissues, and characterized the viral components in the microbiome between lab mice and pet store mice.

## 2. Materials and Methods

### 2.1. Ethical Approval and Informed Consent

All studies were conducted in accordance with the recommendations put forth in the Guide for the Care and Use of Laboratory Animals and were approved by the University of Missouri Institutional Animal Care and Use Committee.

### 2.2. Animals

C57BL/6 mice (JAX, 4 males and 4 females) were directly purchased from Jackson Laboratory (Sacramento, CA, USA and Bar Harbor, ME, USA), C57BL/6NHsd mice (HSD, 4 males, 4 female) from Harlan Laboratory (Harlan Sprague Dawley, Indianapolis, IN, USA) and C57BL/6NTac mice (TAC, 4 males and 4 females) from Taconic (Taconic Biosciences, Inc., La Jolla, CA, USA, IN facilities). Pet store mice were purchased from Petco pet store (PS, 2 males and 2 females) and Columbia Pet Center (2 males and 2 females). All mice were around 4 weeks old and were post-weaning.

### 2.3. Tissue Collection

All mice were euthanized by carbon dioxide asphyxiation and tissues including respiratory tissue (lungs; whole pluck), dermal tissue (glabrous perianal skin), and gastrointestinal tissue (ileum) were collected and flash-frozen in liquid nitrogen and stored in cryovials at −80 °C.

### 2.4. RNA, DNA Extraction and cDNA Synthesis

Qiagen RNeasy kit (Cat #74104) and Qiagen DNeasy blood and tissue kit (Cat #69506) were used for RNA isolation and DNA isolation, respectively. The Agencourt AMPure XP kit (Beckman Coulter, Brea, CA, USA) was applied for cDNA purification after cDNA synthesis. A hundred µL of cDNA was processed by adding 100 µL of the AMPure XP beads. Forty µL of elution buffer was added to dilute the purified cDNA according to the manufacturer’s recommendations. The quality and quantity were measured by Qubit. Only one tissue type was used at a time to prevent cross-contamination of samples.

### 2.5. Primer Information

Primer A: GTTTCCCAGTCACGATANNNNNNNNN a random primer used for cDNA synthesis in the first round and the specific primer B: GTTTCCCAGTCACGATA used for the generated template application. (Primers purchased from Integrated DNA Technologies, Coralville, IA, USA).

### 2.6. Library Preparation

For the sequence library construction preparation, every 4 samples of the same type were pooled in equal volume for a single sequencing library. For instance, 4 DNA samples from the female mice sampled at the skin were pooled together and treated as one single pooling group.

Automated dual-indexed libraries were constructed with 100–250 ng cDNA or gDNA using the KAPA HTP Library Kit (KAPA Biosystems, Wilmington, MA, USA). 250 bp length inserts were targeted by using the SciClone NGS instrument (Perkin Elmer, Waltham, MA, USA). Twenty-four cDNA libraries were pooled pre-capture generating an 18 µg library pool. Twenty-four gDNA libraries were pooled pre-capture generating a 27 µg library pool.

### 2.7. Virome Sequencing

Both library pools were hybridized with a custom Nimblegen probe set (Roche), termed “ViroCap”, targeting a pan-virome space [23]. The concentration of each captured library pool was accurately determined through qPCR (KAPA Biosystems) to produce cluster counts appropriate for the Illumina HiSeq4000 platform. One lane of 2 × 125 sequence data was generated per library pool yielding an average of 4 Gb of data per sample. Viral sequences were identified as previously described [23]. Briefly, both nucleotide and translated sequence alignments were used to identify viral reads, and data were manually reviewed. For sequence coverage determination, we used RefCov (http://gmt.genome.wustl.edu/packages/refcov/ accessed on 15 December 2018). Viral genomes were assembled with IDBA-UD [24], and they were manually reviewed with Tablet [25]. SAMtools [26] was used for sequence alignment evaluation.

### 2.8. Statistical Analysis

Two-way analysis of variance (ANOVA) followed by Holm-Sidak for pairwise multiple comparisons, two factors: sex and source. *p* < 0.05 was considered significant difference.

## 3. Results

### 3.1. Comparison of the Eukaryotic Virome of Mice from Laboratories and Pet Stores

**Figure 1:**
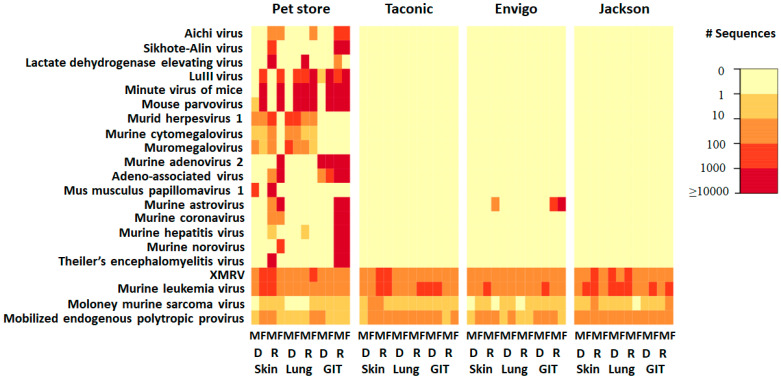
Heat map of vertebrate viruses from individual mice. Mice are grouped according to the source from which they were obtained: Pet Store, Taconic Farms, Envigo and the Jackson Laboratory. RNA (R) and DNA (D) were isolated from samples of gastrointestinal tissue (GIT), lung, and skin from both male (M) and female (F). Viruses detected are on the vertical axis. The value in the color key shows the range of the detected sequence number.

A subjective review of viruses detected in each group of mice revealed two clear patterns. First, pet store (PS) mice harbored a rich virome in multiple tissues while lab mice harbored a limited diversity of eukaryotic viruses. Second, several eukaryotic retroviral sequences were detected in all groups. Twenty-one virus species in total were detected in samples from PS mice, including both DNA and RNA viruses belonging to ten families. Within those 21 species, there were 9 DNA and 12 RNA viruses. As expected, RNA viruses (i.e., *Picornaviridae*, *Arteriviridae*, *Astroviridae*, *Coronaviridae*, and *Caliciviridae*) were detected from sequenced cDNA reverse-transcribed from sample RNA. In contrast, DNA viruses (i.e., *Parvoviridae*, *Herpesviridae*, *Adenoviridae*, and *Papillomaviridae*) were detected in both DNA and RNA extracted from samples, suggesting active replication by many of these viruses. Sequences matching *Retroviridae* were detected in both DNA and RNA from all tissues, suggesting that the former represents proviral DNA within the host genome with some active gene transcription (Figure 1).

Aside from the *Retroviridae*, the remaining viruses detected in pet store mice reflected three different assigned orders, five different unassigned orders, and nine different families of viruses, ranging from *Picornaviridae* to *Caliciviridae* (Table 1).

**Figure 2:**
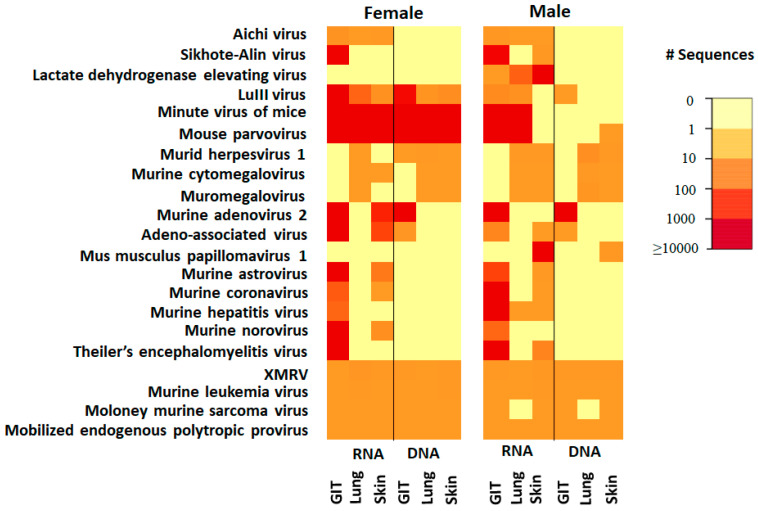
Heat map of vertebrate viruses from pet store mice. Mice are grouped according to the sex including female (**left**) and male (**right**): RNA (R) and DNA (D) were isolated from samples of gastrointestinal tissue (GIT), lung, and skin. Viruses detected are on the vertical axis. The value in the color key shows the range of the detected sequence number.

Analysis of the PS mice based on sex revealed that lactate dehydrogenase elevating virus (LDEV) and mus musculus papillomavirus 1 were only detected in samples from the male mice (Figure 2). In addition, the heat map of vertebrate viruses showed there is difference between sex even among the same source such as pet store mice. Based on our knowledge, there is limited data documented on the characterization of differences on the virome between sex in lab mice.

**Figure 3:**
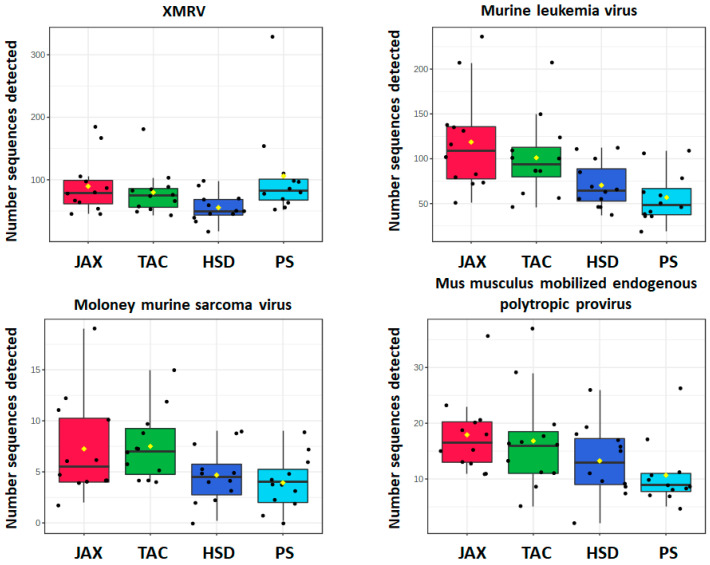
Number of retrovirus sequences detected in mice obtained from different sources: Jackson Laboratory (JAX), Taconic Farms (TAC), Envigo (HSD), and Pet store (PS).

The number of retrovirus sequences detected differed between lab mice and PS mice. The four detected retroviruses included xenotropic murine leukemia virus-related virus (XMRV), murine leukemia virus (MLV), Moloney murine sarcoma virus, and *Mus musculus* mobilized endogenous polytropic provirus. Within the retrovirus, while not absolutely quantitative because the samples are pooled so not evaluated on a per-animal basis and the amplification steps in the protocol can introduce bias. Even so, the number of sequences detected suggested a trend in viral load among the retroviruses comprising greater amounts of XMRV and MLV relative to Moloney murine sarcoma and the polytropic provirus, in both lab mice and PS mice. Two-way ANOVA followed by Holm-Sidak post hoc tests detected significant differences between male JAX and male PS samples, in the number of sequences mapped to MLV (*p* = 0.008) and Moloney murine sarcoma virus (*p* = 0.045) (Figure 3).

Based on the abundance of sequences detected, the six most abundance detected viruses included mouse parvovirus, minute virus of mice, murine adenovirus 2, murine coronavirus, murine hepatitis virus, and Theiler’s encephalomyelitis virus.

### 3.2. Comparison of the Eukaryotic Virome within Laboratory Mice

Within samples from laboratory mice, one difference in the abundance of retrovirus sequences was found between mice from different vendors. Specifically, MLV was detected more frequently in samples from JAX compared to samples from HSD (*p* = 0.037) (Figure 3).

Aside from the *Retroviridae*, astrovirus was the only other virus detected in samples in lab mice. Murine astrovirus was detected in only one of the three lab mouse suppliers (Envigo) (Figure 1).

### 3.3. Comparison of the Viruses in Specific Tissues/Tropisms

From each mouse, samples were collected of respiratory tissue (lungs; whole pluck), dermal tissue (glabrous perianal skin), and gastrointestinal tissue (ileum). The skin is the representative tissue tropic that exposure to the environment at large degree considering the environment is one of the sources that the host was contracted with different virus. The lung is the representative tissue for respiratory organ in the host, and the ileum is the representative tissue from the gastrointestinal which serves as reservoir for intestinal virus infection. Within the more diverse PS virome, tissue tropisms were readily apparent. The recently identified *Mus musculus* papillomavirus, was detected only in skin tissue (Table 2). All three members of the *Herpesviridae* (MHV1, MCMV, and muromegalovirus) were detected in skin and lung, but not the ileum, while murine adenovirus 2 and adeno-associated viruses were detected in the ileal and skin samples, but not the lung. Endogenous retroviruses (ERVs) were present in all tissue types.

### 3.4. Potential for Novel Virus Identification

**Figure 4:**
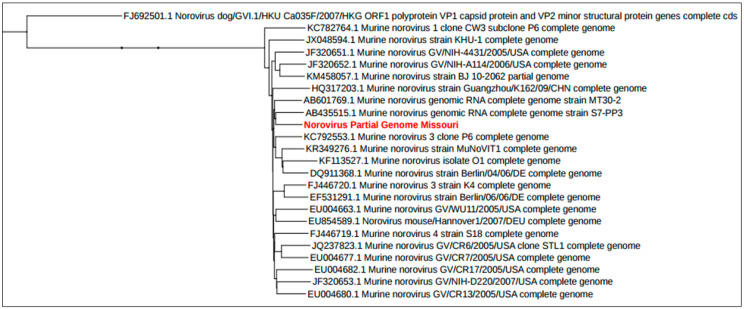
Phylogenetic tree of murine noroviruses. A single representative dendrogram showing the phylogenetic relationship of one potentially unique virus (shown in red) identified in pet store mice (92% nucleotide sequence identity to the next most-closely related strain).

In an effort to identify putative novel viruses, sequence of the norovirus found in PS mice from male gastrointestinal tissue sample and female gastrointestinal tissue and skin sample was compared to known murine noroviruses (Figure 4). This virus was found to share 92% sequence identity with the most-closely related strain found in GenBank. Given that noroviruses are highly mutable RNA viruses, this finding was not surprising, but reinforces that screening tools such as ViroCap can yield data on novel strains that may be worthy of further characterization and pursuit. (BioProject ID: PRJNA733600). The other viruses that were assembled did not differ greatly from previously identified/sequenced genomes.

## 4. Discussion

While the majority of microbiota research focuses on the bacterial component, characterization of the eukaryotic virome has lagged due to the lack of efficient methods to comprehensively survey viromes. Viruses, unlike bacteria, lack a universal conserved gene (such as the 16S rRNA gene in bacteria) enabling the identification and classification of different community members based on variable regions within that conserved gene. Virome identification, on the other hand, is complicated by the large diversity of viral genomes which do not share any universal phylogenetic marker, can be made from RNA or DNA, and can vary greatly in size and structure [27,28]. While shotgun metagenomic sequencing has been used to identify eukaryotic viral sequences, capture-based methods like ViroCap enrich the pool of nucleic acid for viral DNA and RNA prior to sequencing, and allows investigation of the eukaryotic virome in greater detail [23]. Importantly, because the probes in ViroCap tile the complete genomes of the targets, we are subsequently able to carry out comparative analysis of the enriched, sequenced genomes. Our results are in agreement with previous reports regarding the differences in virome composition between laboratory, pet store [29,30] and wild mice [31,32] and confirm that while most murine viral pathogens have been eradicated from lab mouse production facilities, these agents are abundant in non-laboratory populations [33].

The virome in mammalian hosts includes prokaryotic viruses (bacteriophages) that infect resident bacteria, eukaryotic viruses which transiently infect the host cells, and viral elements including retroviruses that are integrated into the host genome [34]. There is increasing evidence of a relationship between the eukaryotic virome and susceptibility to immune-mediated diseases such as inflammatory bowel disease (IBD) [35] and rheumatoid arthritis [36,37]. Furthermore, studies on the interaction between viruses and bacteria suggest direct inter-kingdom communication, and synergistic influences on the development of host immunity and susceptibility to various conditions [38]. The eukaryotic virome as a key component of the virome likely plays a critical role in host health and disease, including unidentified, subclinical viruses which may influence host physiology, immune system development, and disease/model susceptibility. As a consequence, there are potential influences on preclinical research investigating disease mechanisms, and development of novel therapeutics. All of these issues highlight the importance of a deeper understanding of the eukaryotic virome of mouse models [39,40].

To optimize mouse models of disease, a better understanding of the role of the microbiome, including the virome, in model phenotypes is needed. However, the variability in endogenous retroviruses remain. This finding was not unexpected given recent studies by Lee et al. [41] that used a TREome probe from murine leukemia virus-type endogenous retroviruses to survey C57BL/6J mice. They noted marked variability in the MLV-ERV landscape that depended on several factors, including individual mouse, sex, tissue, and cell type. What remains to be determined is the impact of such variation on individual mice as well as mouse models of disease in general.

Our study also identified murine astrovirus in both laboratory and pet store mice. Murine astrovirus was first found in nude mice in 1985, followed by the complete genome sequence from a wild mouse in 2011 [42,43]. The first complete murine astrovirus genome sequence that obtained from immunocompetent lab mice and published in 2012 [44]. Subsequent reports have confirmed the existence of astrovirus in laboratory mice [44,45], but the true prevalence in most research colonies remains unknown as it is not on many health monitoring profiles.

It has been speculated that the high prevalence of murine astrovirus in lab mice coupled with the diversity of virus strain [45] and asymptomatic infection could contribute to phenotypic differences between mice used in research.

This study also identified a murine norovirus in pet store that shared 92% nucleotide sequence identity to the next most-closely related strain. Murine noroviruses have been proposed as model agents for the study of human noroviruses [46,47]. However, unlike their human counterparts, which are a leading cause of non-bacterial epidemic gastroenteritis, murine noroviruses are asymptomatic unless infections occur in mice lacking anti-viral defense mechanisms [48]. However, their study has revealed novel putative roles for these viruses in intestinal homeostasis. For example, germ-free mice infected with MNV have increased the numbers of CD4^+^, CD8^+^ T cells and IFN-γ when compared to norovirus-free mice [49]. To this end, MNV-CR6 infection suppresses the expansion of group 2 innate lymphoid cells, a function similar to that of commensal bacteria [50]. In addition, MNV-CR6 infection of antibiotic-treated mice protected against DSS-induced intestinal injury. These findings suggest that noroviruses may play a physiological beneficial role in intestinal homeostasis. The identification of additional noroviruses such as that identified in this study provides further tools to understand the complex role of this family of viruses in health and disease.

## 5. Conclusions

Characterization of the microbiome of lab mice, pet store mice and wild mice stands to greatly aid our understanding of the crucial roles the microbiome play in host physiology and disease. Moreover, ensuring that the murine microbiome is representative of that seen in humans can yield more informative and translational mouse models of disease. Critical to this characterization and refinement is inclusion of the virome in discussions of the microbiome. Because lab mice are relatively free of viral pathogens, inclusion of studies of pet store or wild mice is needed to better incorporate the role of viruses. Collectively, such studies will also enhance our understanding of inter-kingdom interactions between viral and bacterial communities and the host.

## Figures and Tables

**Table 1:** Categorization of viruses identified (retroviruses excluded). Table shows the summary of the identified viruses that belong to different viral families based on the common taxonomy for the classification of viruses. Besides the retrovirus family that was shared by both pet store mice and lab mice, the listed 9 viral families in this table were only found in pet store mice. * Astrovirus as the only virus found in lab mice.

DNA/RNA Virus	Family	Virus Species
RNA	Picornaviridae	Aichi Virus
RNA	Picornaviridae	Skihote alin virus
RNA	Picornaviridae	Theilers encephalomyelitis virus
RNA	Arteriviridae	Lactate dehydrogenase elevating virus
RNA	Astroviridae *	Murine astrovirus
RNA	Coronaviridae	Murine coronavirus
RNA	Coronaviridae	Murine hepatitis virus
RNA	Caliciviridae	Murine norovirus
DNA	Parvoviridae	Lull virus
DNA	Parvoviridae	Minute virus of mice
DNA	Parvoviridae	Mouse parvovirus
DNA	Parvoviridae	Adeno associated virus
DNA	Herpesviridae	Murid herpesvirus 1
DNA	Herpesviridae	Murine cytomegalovirus
DNA	Herpesviridae	Muromegalovirus
DNA	Adenoviridae	Murine adenovirus 2
DNA	Papillomaviridae	*Mus musculus* papillomavirus. Type 1

**Table 2:** Tissue specificity vertebrate viruses in pet store mice and laboratory mice.

Tissue Specificity Vertebrate Virus in Pet Store Mice				
Viral Family	Viral Species	GI	Lung	Skin
Retroviridae	XMRV	4/4 (100%)	4/4 (100%)	4/4 (100%)
Retroviridae	Murine leukemia viruses	4/4 (100%)	4/4 (100%)	4/4 (100%)
Retroviridae	Moloney murine sarcoma virus	1/4 (25%)	1/4 (25%)	2/4 (50%)
Retroviridae	*Mus musculus* mobilized endogenous polytropic provirus	4/4 (100%)	4/4 (100%)	4/4 (100%)
Astroviridae	Murine astrovirus	2/4 (50%)	0/4 None	2/4 (50%)
Picornaviridae	Aichi Virus	2/4 (50%)	1/4 (25%)	2/4 (50%)
Picornaviridae	Skihote alin virus	2/4 (50%)	0/4 None	1/4 (25%)
Arteriviridae	Lactate dehydrogenase elevating virus	1/4 (25%)	1/4 (25%)	1/4 (25%)
Parvoviridae	LuIII virus	3/4 (75%)	3/4 (75%)	2/4 (50%)
Parvoviridae	Minute virus of mice	3/4 (75%)	3/4 (75%)	2/4 (50%)
Parvoviridae	Mouse parvovirus	3/4 (75%)	3/4 (75%)	2/4 (50%)
Herpesviridae	Murid herpesvirus 1	0/4 None	4/4 (100%)	3/4 (75%)
Adenoviridae	Murine adenovirus 2	4/4 (100%)	0/4 None	1/4 (25%)
Parvoviridae	Adeno associated virus	4/4 (100%)	0/4 None	2/4 (50%)
Papillomaviridae	*Mus musculus* papillomavirus. Type 1	0/4 None	0/4 None	2/4 (50%)
Coronaviridae	Murine coronavirus	2/4 (50%)	0/4 None	2/4 (50%)
Coronaviridae	Murine hepatitis virus	2/4 (50%)	0/4 None	1/4 (25%)
Caliciviridae	Murine norovirus	2/4 (50%)	0/4 None	1/4 (25%)
Picornaviridae	Theilers encephalomyelitis virus	2/4 (50%)	0/4 None	1/4 (25%)
**Tissue Specific Vertebrate Viruses in Laboratory Mice**				
Retroviridae	XMRV	12/12 (100%)	12/12 (100%)	12/12 (100%)
Retroviridae	Murine leukemia viruses	12/12 (100%)	12/12 (100%)	12/12 (100%)
Retroviridae	Moloney murine sarcoma virus	6/12 (50%)	8/12 (67%)	8/12 (67%)
Retroviridae	*Mus musculus* mobilized endogenous polytropic provirus	12/12 (100%)	11/12 (92%)	12/12 (100%)
Astroviridae	Murine astrovirus	2/12 (17%)	0/12 None	1/12 (8%)

## Data Availability

The data supporting reported results can be found in this manuscript.
